# Response to hemophilia B gene therapy based on adenoassociated virus serotype 5 antibody status

**DOI:** 10.1016/j.rpth.2026.103447

**Published:** 2026-03-26

**Authors:** Wolfgang Miesbach, Alok Srivastava

**Affiliations:** 1 Medical Clinic 2, University Hospital Frankfurt, Frankfurt, Germany; 2 St. John’s Research Institute & St. John’s Medical College Hospital, Bengaluru, India

We read with great interest the article by Klamroth et al. [1] on the 4-year data of participants with pre-existing adenoassociated virus serotype 5 (AAV5)-neutralizing antibodies (NAb^+^) enrolled in the HOPE-B gene therapy trial of etranacogene dezaparvovec for hemophilia B. The simultaneous publication of HOPE-B subgroup analyses stratified by AAV5 NAb status [1,2] now provides the first sufficiently robust within-trial comparison of NAb^+^ (*n* = 21) vs NAb^−^ (*n* = 33) participants across a clinically meaningful NAb titer range (8.5-678; *n* = 20; 3212, *n* = 1).

Pre-existing AAV NAbs were long considered an exclusion criterion for AAV-based gene therapy because they were expected to coat the viral capsid; stearically block its receptor-binding domains; and thereby prevent cell attachment, endocytosis, and nuclear delivery of the transgene [3]. This concept was reinforced by standard *in vitro* neutralization assays, in which even low-titer serum antibodies, with the sensitivity of assays available at that time, consistently inhibited AAV transduction of cultured cells in a dose-dependent manner, with ≥50% transduction inhibition typically occurring at serum dilutions of 1:10 to 1:100, leading to the assumption that any detectable NAbs would similarly abrogate *in vivo* gene transfer [4].

The initial evidence challenging this paradigm emerged from a small but provocative phase 2b trial. Von Drygalski et al. [5] reported, in 2019, 3 participants with severe hemophilia B and pre-existing AAV5 NAbs (screening titers, 25-48; mean, 39), who achieved sustained endogenous factor (F)IX-Padua production in the high mild to nonhemophilia range over 5 years [6]. Critically, these titers were relatively low, and the magnitude of benefit in antibody-positive patients across a broader titer range remained incompletely characterized.

In contrast to earlier studies that excluded patients with detectable AAV5 NAbs, the subsequent phase 3 HOPE-B trial [7] enrolled participants irrespective of AAV5 NAb status, with NAb^+^ titers ranging from 8.5 to 3212, enabling within-trial comparison of long-term outcomes [1,2]. Baseline demographics and disease characteristics were comparable between NAb^−^ and NAb^+^ participants [1,2] (Table 1). For NAb^+^ status (*n* = 21), FIX activity data at year 4 were available for 15 participants (4 excluded: 1 liver transplant performed at month 28; 3 missing samples or contaminated due to concurrent exogenous FIX use; 1 patients with high titer anti-AAV antibodies; and 1 patient who only revieved 10% of the dosage).

**Table 1:** Baseline demographics and efficacy/safety outcomes compared between NAb-positive [1] and NAb-negative [2] participants from the HOPE-B trial.

Characteristics	NAb^+^ (*n* = 21)	NAb^−^ (*n* = 33)
Age (y), mean ± SD (range)	44.5 ± 17.5 (19-75)	39.5 ± 14.5 (21-73)
Severe hemophilia B: FIX ≤ 1 IU/dL (%)	76.2	85
Moderately severe: FIX 1-2 IU/dL (%)	23.8	15
Prior hepatitis C infection (%)	66.7	52
Prior hepatitis B infection (%)	23.8	12
HIV-positive status (%)	4.8	6
FIX activity (IU/dL), mean ± SD^a^	34.0 ± 16	39.0 ± 16.8
FIX activity, median (range)^a^	33.0 (8-61)	35.7 (4.7-80.1)
ABR reduction (%)^b^^,^^c^	74	85
Infusion-related reactions, *n* (%)^d^	5 (23.8)	2 (6.1)
ALT elevation, *n* (%)	3 (14.3)	6 (18)

ABR, annualized bleeding rate; ALT, alanine aminotransferase; F, factor; NAb, neutralizing antibody.

a For NAb^+^, FIX activity data at year 4 available for 15 participants (3 excluded: 1 liver transplant at month 28; 2 nonresponders [1 with highest NAb titer 3212; 1 who received ∼10% of intended dose]; 3 with missing/contaminated samples due to concurrent exogenous FIX use).

b ABR comparison between lead-in period (≥6 mo on continuous prophylaxis) and months 7-48 postgene therapy.

c NAb^+^, represents responder analysis set (*n* = 19); 2 nonresponders excluded (remained on continuous FIX prophylaxis).

d Fourfold higher incidence in NAb^+^ vs NAb^−^, participants; 4 of 5 NAb^+^, infusion-related reactions occurred in patients with NAb titers ≥ 198.

Efficacy outcomes were essentially equivalent: at 4 years, mean endogenous FIX activity was 39.0 ± 16.8 IU/dL in NAb^−^ participants (*n* = 33) and 34 ± 16 IU/dL in NAb^+^ responders (*n* = 15). Approximately 50% of each cohort achieved FIX levels of ≥40 IU/dL. Notably, no clinically meaningful correlation between individual AAV5 titer at baseline and the median endogenous FIX activity from month 7 to 48 was identified.

Bleeding protection was substantial in both cohorts. The annual bleeding rate (ABR) was compared between lead-in and months 7 to 48 postgene therapy. Adjusted annual bleeding rate (months 7-48) decreased 85% (from 3.80 to 0.57 bleeds/y) in NAb^−^ participants and 74% (from 4.64 to 1.18) in NAb^+^ responders, with near elimination of spontaneous and joint bleeding in both groups [1,2]. No NAb^−^ participant returned to continuous FIX prophylaxis over 4 years; only 1 of 19 NAb^+^ responders resumed prophylaxis at month 29 to 30 (5.3%). One NAb^+^ participant with a screening titer of 3212 failed to respond, achieving only 1 to 1.5 IU/dL endogenous FIX and remaining dependent on continuous prophylaxis [1].

All 21 NAb^+^ participants included in safety analyses. Safety profiles diverged in clinically important ways. Infusion-related reactions (IRRs) occurred substantially more frequently in NAb^+^ than those in NAb^−^ participants: 23.8% (5/21) vs 6.1% (2/33), representing nearly 4-fold increased risk [1,2]. Four of 5 NAb^+^ IRRs occurred in patients with titers of ≥198, suggesting a titer-dependent relationship.

All IRRs were managed with standard measures—observation, temporary infusion slowing, antihistamines, and single-dose cortisone. One participant with a titer of 198.9 received only ∼10% of the planned dose owing to a moderate hypersensitivity reaction and did not achieve therapeutic FIX expression. All other participants with IRRs achieved sustained FIX expression, indicating that manageable IRRs do not constitute a biological barrier to transduction.

Hepatic safety and long-term tolerability were similar. Alanine aminotransferase elevations occurred with similar frequency in both cohorts—18% (6/33) in NAb^−^ and 14.3% (3/21) in NAb^+^ participants—confined to the first 3 months postinfusion [1,2]. Reactive corticosteroid treatment (median duration, ∼80 days) did not compromise efficacy; participants who had achieved FIX expression at steroid initiation maintained stable transgene-derived FIX activity through 4 years. There were no cases of late or persistent hepatotoxicity, no FIX inhibitors, and no thrombotic events. Radiologic assessment of the liver on long-term follow-up will further confirm absence of toxicity. Two serious adverse events in year 4 showed no evidence of AAV5 vector DNA integration and were deemed unrelated to treatment [1,2].

These data support reliable efficacy at baseline AAV5 NAb titers up to 678, whereas the upper boundary between this range and the single nonresponder at 3212 remains undefined (Table 2). The ongoing trial, NCT06003387, which enrols participants with baseline AAV5 NAbs across a wider titer spectrum, will be essential to guide clinical practice [8].

**Table 2:** Titer-dependent efficacy and IRRs after etranacogene dezaparvovec in NAb^+^ study participants.

Anti-AAV titer range	Evidence [1,5]	Efficacy	Safety
<198	Phase 2b (*n* = 3) + HOPE-B NAb^+^ (*n* = 16)	Established	IRR, ∼5% (1/19)
198-678	HOPE-B (*n* = 5)	Established	IRR, 80% (4/5); manageable
>678–3212	None	Unknown	Unknown
≥3212	Single nonresponder	Ineffective	IRR; no late safety problems

IRR, infusion-related reaction; NAb^+^, positive AAV neutralizing antibody.

It should be noted though that these interpretations of titer levels are only applicable to this product using the specific methodology used for the assays along with the vector doses administered in this clinical trial. In the absence of universal standards of anti-AAV antibody assays, the comparability of titers using different assays methods, reagents, and products is a challenge [9]. The lack of calibration and comparative data between different assay methods for quantification of antibody titers against different products and their possible clinical impact is a gap, which needs to be addressed.

In conclusion, these comparative data demonstrate that pre-existing AAV5 NAbs are not a barrier to successful etranacogene dezaparvovec gene therapy. Key limitations include the modest NAb^+^ cohort (*n* = 15), lack of standardized titer assays, and titer-dependent infusion-related reactions. The ongoing NCT06003387 trial will be essential to establish clinical applicability and evidence-based screening protocols for NAb^+^ candidates.
